# Use of disease assessment tools to increase the value of case reports on Susac syndrome: two case reports

**DOI:** 10.1186/s13256-023-03838-9

**Published:** 2023-04-13

**Authors:** Danielle R. Bullock, Robert T. Spencer, Richard K. Vehe, Sunil Srivastava, Robert M. Rennebohm

**Affiliations:** 1 Division of Pediatric Rheumatology, Department of Pediatrics, University of Minnesota, East Bldg Rm M668, 2450 Riverside Ave, Minneapolis, MN 55454 USA; 2 Colorado Arthritis Center, Rheumatology, 401 E Hampden Ave, Suite 410, Englewood, CO 80113 USA; 3 Ophthalmology Department, Cole Eye Institute, Cleveland Clinic, 2022 E 105th St, Cleveland, OH 44106 USA; 4 Division of Pediatric Rheumatology, Institute of Pediatrics, Formerly at the Cleveland Clinic, 9500 Euclid Ave, Cleveland, OH 44195 USA

**Keywords:** Susac syndrome, Disease assessment tools, Clinical course, Outcome, Case report

## Abstract

**Background:**

Susac syndrome is an immune-mediated, ischemia-producing, occlusive microvascular endotheliopathy that threatens the brain, retina, and inner ear. There is a need for disease assessment tools that can help clinicians and patients to more easily, accurately, and uniformly track the clinical course and outcome of Susac syndrome. Ideally, such tools should simultaneously facilitate the clinical care and study of Susac syndrome and improve the value of future case reports. To meet this need, two novel clinical assessment tools were developed: the Susac Symptoms Form and the Susac Disease Damage Score. The former is a comprehensive self-report form that is completed by patients/families to serially document the clinical status of a patient. The latter documents the extent of damage perceived by individual patients/families and their physicians. Both forms were initially trialed with two particularly representative and instructive patients. The results of this trial are shared in this report.

**Case presentation:**

Patient 1 is a 21-year-old Caucasian female who presented with an acute onset of headache, paresthesias, cognitive dysfunction, and emotional lability. Patient 2 is a 14-year-old Caucasian female who presented with an acute onset of headache, cognitive dysfunction, urinary incontinence, ataxia, and personality change. Both patients fulfilled criteria for a definite diagnosis of Susac syndrome: both eventually developed brain, retinal, and inner ear involvement, and both had typical “snowball lesions” on magnetic resonance imaging. The Susac Symptoms Form documented initial improvement in both patients, was sufficiently sensitive in detecting a subsequent relapse in the second patient, and succinctly documented the long-term clinical course in both patients. The Disease Damage Score documented minimal disease damage in the first patient and more significant damage in the second.

**Conclusions:**

The Susac Symptoms Form and the Disease Damage Score are useful disease assessment tools, both for clinical care and research purposes. Their use could enhance the value of future case reports on Susac syndrome and could improve opportunities to learn from a series of such reports.

**Supplementary Information:**

The online version contains supplementary material available at 10.1186/s13256-023-03838-9.

## Background

Susac syndrome (SuS) is an immune-mediated, ischemia-producing, occlusive microvascular endotheliopathy that threatens the brain, retina, and inner ear [1–3]. In its most classic form it is characterized by the clinical triad of encephalopathy, branch retinal artery occlusion (BRAO), and sudden low-frequency hearing loss, and by the magnetic resonance imaging (MRI) finding of “snowball lesions” in the central portion of the corpus callosum. Disease severity falls along a spectrum, ranging from mild and fully reversible ischemic dysfunction to permanent devastating damage from microinfarction [3].

SuS primarily affects young women between the ages of 20–40 years, but similarly aged men can be affected, as can children and patients in their 40s and 50s [1]. The female:male ratio is approximately 3:1 [1]. Data regarding incidence and prevalence are limited. The annual incidence reported in Austria is 0.024/100,000 [4]. However, a recent study suggests that the annual incidence in Israel is at least 5.4 times that in Austria [5]. The ethnicity of patients reported in the medical literature suggests that SuS occurs most commonly in Caucasians and least commonly in Asians and Africans.

Although SuS is relatively rare, it needs to be considered in the differential diagnosis of any patient who presents with unexplained persistent headache, unexplained encephalopathy, unexplained visual loss, or unexplained acute hearing loss, because any one component of the Susac clinical triad can be the sole presenting feature. SuS has become newly relevant during the coronavirus disease 2019 (COVID-19) pandemic because the ischemia-producing, occlusive microvascular endotheliopathy of SuS [6–8] might serve as a model for aspects of the ischemia-producing, occlusive microvascular endotheliopathy seen in COVID-19 and in spike protein-mediated complications of COVID vaccines [9]. Indeed, SuS has been documented to occur in the wake of COVID-19 [10], and an official World Health Organization (WHO) database has reported BRAO, encephalopathy, and tinnitus as possible adverse events post-COVID vaccination. [11]

Because the immunopathogenesis and the spectrum of clinical courses of SuS appear to be similar to that of juvenile dermatomyositis (JDM), the treatment of SuS has been modeled after treatment of JDM, as have anticipations regarding clinical course [3]. (With both SuS and JDM, patients follow one of three clinical courses: monocyclic, polycyclic, or chronic continuous.) As with JDM, a key to good outcome in SuS is early, appropriately aggressive, appropriately anticipatory, and appropriately sustained immunosuppression. As with any chronic autoimmune disorder, serial assessment of the extent of disease activity not only serves as the basis for adjusting treatment in real time, but also facilitates clinical research.

A major difficulty regarding the treatment and study of SuS has been the dearth of objective biomarkers and the absence of other tools to serially assess extent of disease activity. Unlike JDM and lupus, where a variety of lab tests are available to serially determine disease activity, there currently are no lab tests to serve as reliable biomarkers of disease activity in SuS. Unlike in JDM and rheumatoid arthritis, where physical exam findings serve to guide treatment, physical exam is of limited value in the serial assessment of SuS. Although serial MRI is helpful during the early weeks of illness, serial MRI is of limited value in the long-term follow-up management of SuS. Fluorescein angiography (FA) is an excellent biomarker of SuS disease activity, but FA reflects only what is transpiring in the retinal microvasculature and may or may not reflect what is simultaneously occurring in the microvasculature of the brain and inner ear.

This lack of reliable biomarkers renders the clinician and patient dependent on careful interval history taking. At each clinical encounter, it is necessary to painstakingly collect detailed information about each of many potential symptoms, particularly neurological symptoms, then the severity of those symptoms must be compared with similar detailed narrative assessments on past visits to determine trends and interpret the extent of disease activity. This task is complicated by the fact that it is often difficult to discern whether a given symptom is due simply to disease damage, to ongoing inadequately suppressed disease activity, to as-yet-incomplete recovery from reversible injury, or to a combination of these possibilities. Furthermore, physical reactions to emotional stresses can mimic many symptoms of SuS (for example, headaches), and side effects from medications can also complicate interpretation of a patient’s status.

To help clinicians and patients more easily, accurately, and uniformly track and interpret the patient’s clinical course, we have developed a set of disease assessment tools, the most important of which is the comprehensive Susac Symptoms (SuSx) Form. This self-report form is primarily intended for frequent prospective completion by patients/families to serially document the patient’s clinical status. We hypothesized that data generated by the serially completed forms would facilitate recognition of a patient’s clinical trajectory and would in turn help guide treatment. A companion form, the Susac Disease Damage Score (DDS), is completed periodically by the patient/family and the physician to document the extent of damage.

### Susac Symptoms (SuSx) Form

The SuSx Form was developed with input from several patients. It is a comprehensive, 29-item, patient/family self-report disease assessment tool which uses 100 mm visual analogue scales to capture the extent of 13 neurologic symptoms, 5 inner ear symptoms, 4 eye symptoms, functional difficulties, and impaired quality of life (QOL). It also captures the extent to which the family thinks the disease is still active and has caused damage. (See Additional file 1 for a blank version of the SuSx Form.) The SuSx Form is completed by the patient/family on the day of follow-up clinic visits, at regular intervals between clinic visits, and on an as-needed basis to capture exacerbations and fluctuations of symptoms. A score of zero for an individual item means that the patient is not experiencing that symptom; a score of 100 means the symptom is being experienced to an extremely severe degree. The form is accompanied by a document entitled “Definitions and Gradations for the SuSx Form” (Additional file 2), which defines each symptom and provides examples of mild, moderate, severe, and extremely severe degrees of each. The worst possible Total Susac Symptoms Score is 2200. The worst possible subtotal scores for the neurologic, inner ear, and eye symptoms are 1300, 500, and 400, respectively. In addition to calculating total and subtotal scores, mean scores may be calculated, which is helpful, for example, when fewer than all 13 neurologic symptoms are assessable.

Although this disease assessment tool has not yet been statistically validated, it has exhibited face validity when used in clinical practice to quantitatively determine the trend of a patient’s symptoms. While it is most valuable when used prospectively, it may also be used retrospectively to reconstruct and depict a patient’s clinical course. It is also designed to generate data for research purposes.

### Susac disease damage score (DDS)

The Susac DDS (Additional file 3) is a 28-item form that enables patients/families and their physicians to indicate the extent to which they think the patient has sustained damage to the brain, inner ears, and eyes. The DDS is also accompanied by its own “Definitions and Gradations” document (Additional file 4), which defines each potential form of damage and provides examples of mild, moderate, severe, and extremely severe degrees of each. This disease assessment tool was primarily developed to provide a uniform way of documenting the outcome of individual patients and also helps the clinician determine how much of a patient’s SuSx score might be due to irreversible damage as opposed to ongoing active disease. The worst possible total DDS is 2800–1700 for neurologic damage, 600 for inner ear damage, and 500 for eye damage. A score of zero means no damage is apparent.

After developing these two disease assessment tools, we trialed them with two particularly representative and instructive patients. In this article, we present the SuSx Form and the DDS, and, via detailed case reports, we demonstrate their utility in documenting the clinical presentation, clinical course, response to treatment, and outcome of the two patients. The case reports also demonstrate how data generated by serial use of these forms can increase the value of individual case reports on SuS and can improve opportunity to learn from a future series of such reports.

## Case reports

### Patient 1

The first patient, a 21-year-old Caucasian female, was well until she acutely developed paresthesias in her fingers, mouth, and lips. Over the next week, she also noted fatigue and “started to forget things, often failing to follow-up on things.” These symptoms persisted and gradually worsened. On day 11 of her illness, she developed more dramatic and obvious behavioral abnormalities. She became inappropriately “giddy and giggly.” She seemed unaware of her abnormal behavior. She developed headache, vomiting, and became mildly incoherent. These symptoms prompted emergency admission on Day 12, whereupon the following symptoms were evident:

- Headaches, with vomiting
- Decreased mental alertness, slow thought processing.
- Short-term memory difficulty
- Confusion, disorientation, odd behaviors, compromised insight regarding her condition.
- Decreased executive function (disorganized, not able to take care of her affairs or make good decisions)
- Personality change
- Emotional lability
- Marked inability, intellectually, to do her usual college work.
- Paresthesias
- Difficulty walking
- Imbalance
- Bladder dysfunction
- Apraxia
- Vertigo
- She had no hearing loss, tinnitus, or convincing visual symptoms.

**Fig. 1:**
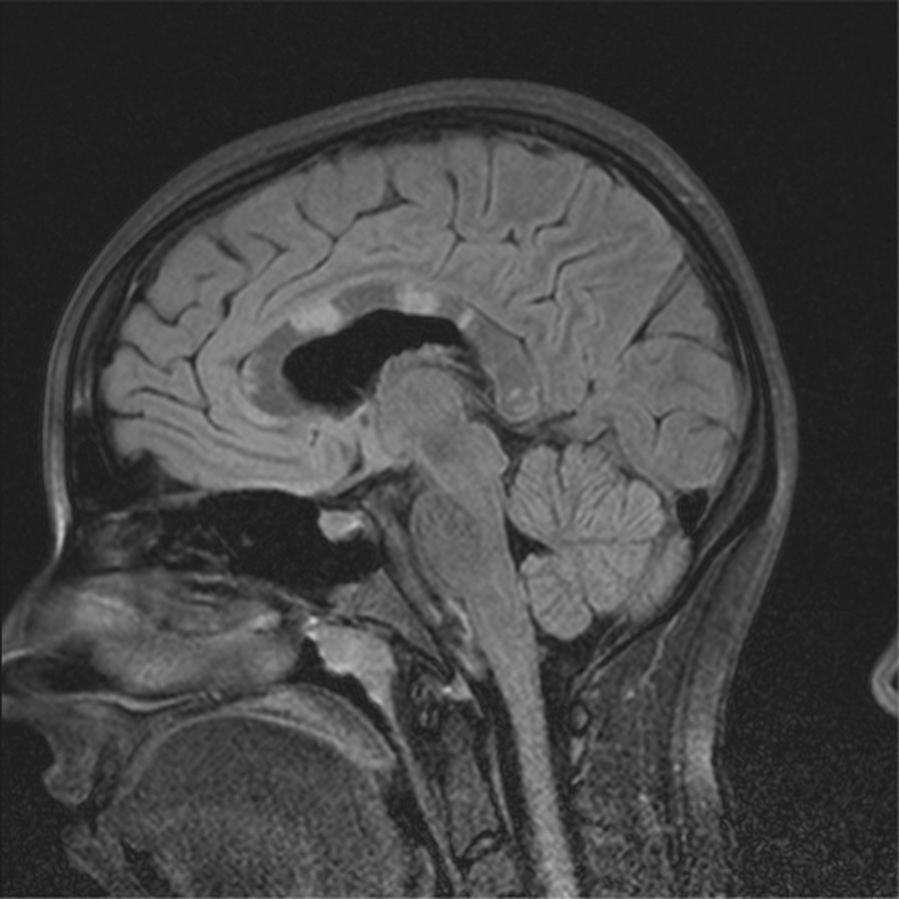
Corpus callosal lesions, case 1

MRI on admission revealed several “snowball” lesions in her corpus callosum, as well as several scattered smaller lesions elsewhere in her white matter (Fig. 1). Although her diagnosis was unclear at the time, she was treated with four consecutive daily pulses of methylprednisolone (1000 mg each), starting on day 13 of her illness. She improved rapidly and considerably. Four days later, she was discharged on oral prednisone 60 mg every morning.

**Fig. 2:**
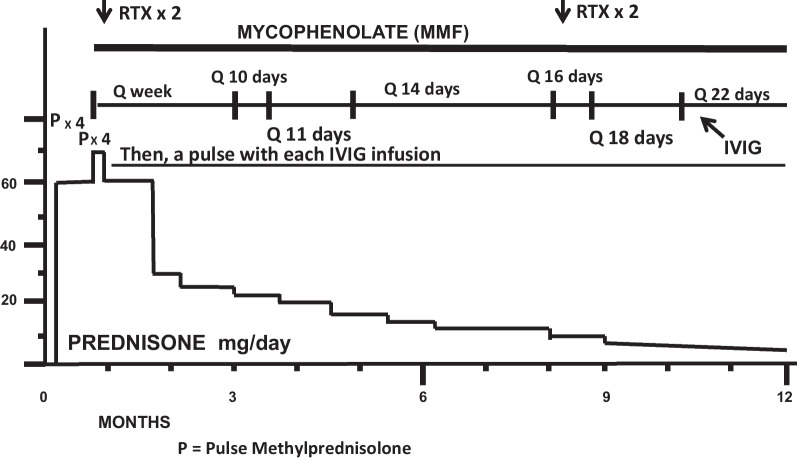
Immunosuppressive treatment: case 1, year 1

**Fig. 3:**
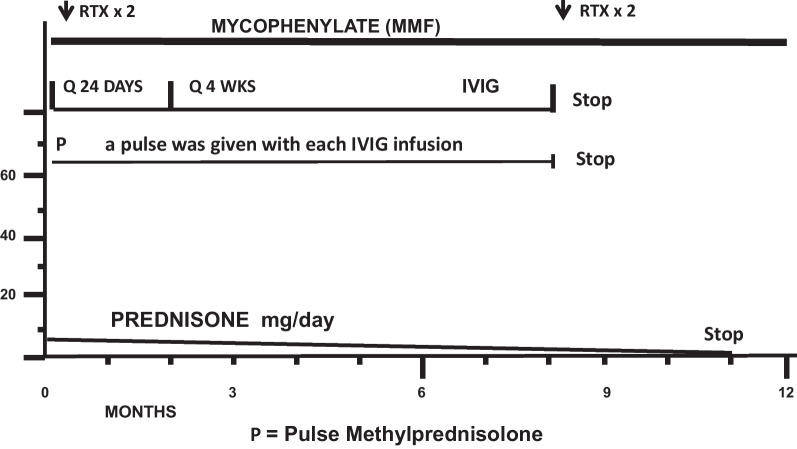
Immunosuppressive treatment: case 1, year 2

**Fig. 4:**
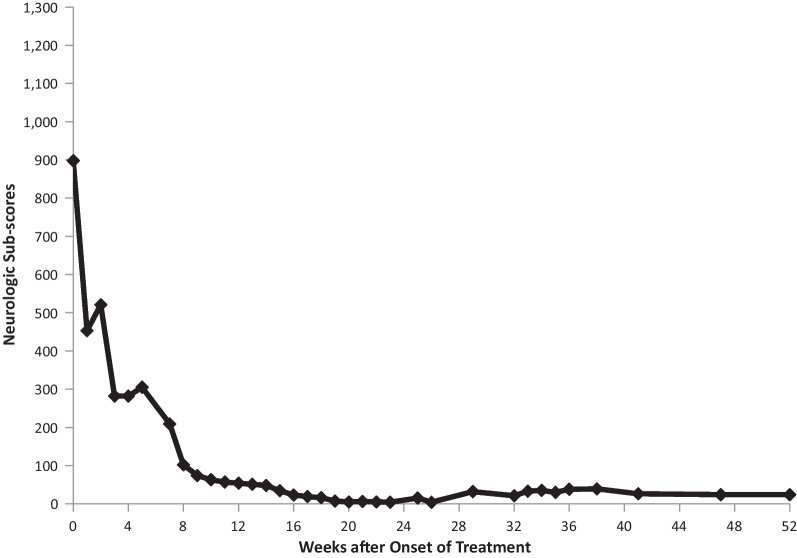
Serial neurologic sub scores: case 1

**Fig. 5:**
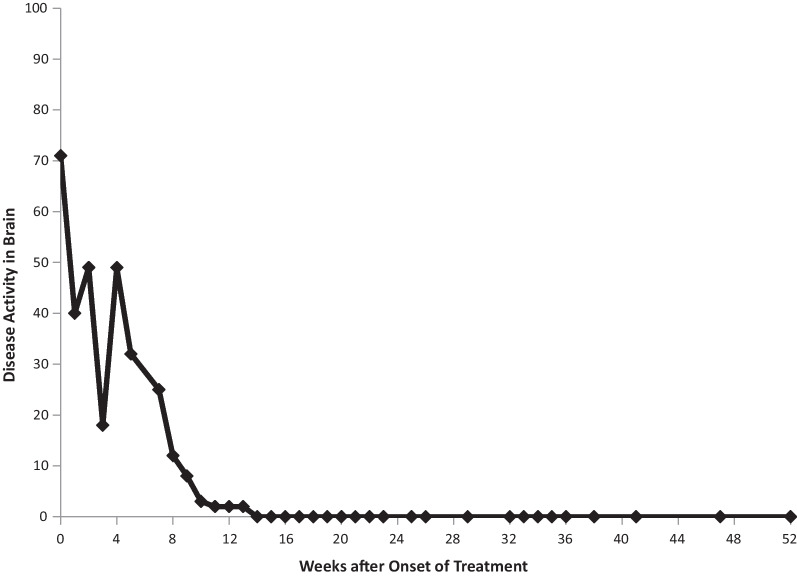
Serial disease activity scores: case 1

Her subsequent treatment, clinical course, and outcome (23 months after onset of treatment) are summarized in Figs. 2, 3, 4, 5, and Tables 1, 2, 3, 4, 5, and 6. In short, she steadily improved, has followed a monocyclic course, and has had an excellent outcome at 23 months after onset of treatment.

**Table 1:** Serial SuSx Scores

	Year 1	Week:	0	1	2	3	4	5	7	8	9	10	11
Patient 1													
*Susac symptoms scores:*													
Neurologic subscore													
Mean score (0–100*)			69	34.8	40.1	21.7	21.7	23.5	16.1	7.8	5.7	4.8	4.4
Sub Total (0–1300)			898	453	521	282	282	305	209	102	74	63	57
Ear subscore													
Mean score (0–100)			9.8	7.8	8.2	5	5.8	7	3.2	2	0.6	0.6	0
Sub total (0–500)			49	39	41	25	29	35	16	10	3	3	0
Eye subscore													
Mean score (0–100)			0	2	13.5	0	0	21.8	3.8	3.8	1.8	1.8	1.3
Sub total (0–400)			0	8	53	0	0	87	15	15	7	7	5
Total symptoms score (0–2200)			947	500	615	307	311	432	240	127	84	73	62
Difficulty performing ADL (0–100)			75	41	48	31	26	18	19	3	3	3	2
Difficulty performing job (0–100)			85	75	75	48	40	33	30	25	20	15	14
Diminished QOL (0–100)			100	60	71	55	50	42	40	36	27	27	27
Total score for above 3 (0–300)			260	176	194	134	116	93	89	64	50	45	43
Oxford Scale (0–6) Overall QOL (100 is best possible score)			–	–	–	90	85	90	90	91	90	89	91
Disease activity:													
Brain (0–100)			71	40	49	18	49	32	25	12	8	3	2
Ears (0–100)			33	35	0	0	0	3	1	0	0	0	0
Eyes (0–100)			0	0	48	11	40	55	15	14	12	10	8

*Week* weeks after onset of treatment, *ADL* Activities of daily living, *QOL* Quality of life

*The second number in all parentheses is the worst possible score (except for QOL)

**Table 2:** Serial SuSx Scores

	Year 1	Week:	12	13	14	15	16	17	18	19	20	21	22	23
Patient 1														
*Susac symptoms scores:*														
Neurologic subscore														
Mean score (0–100)			4.2	3.9	3.7	2.6	1.8	1.5	1.2	0.5	0.4	0.5	0.4	0.3
Sub total			54	51	48	34	23	19	16	7	5	6	5	4
Ear subscore														
Mean score (0–100)			0	0	0	0	0	0	0	0	0	0	0	0
Sub total			0	0	0	0	0	0	0	0	0	0	0	0
Eye subscore														
Mean score (0–100)			1.3	1	0.75	0.5	0	0	0	0	0	0	0	0
Sub total			5	4	3	2	0	0	0	0	0	0	0	0
Total symptoms score			59	55	51	36	23	19	16	7	5	6	5	4
Difficulty performing ADL (0–100)			2	2	2	2	2	2	2	2	2	2	0	0
Difficulty performing job (0–100)			14	14	14	10	10	10	8	5	5	5	4	4
Diminished QOL (0–100)			27	25	23	20	20	16	15	13	11	10	8	8
Total score for the above 3 items			43	41	39	32	32	28	25	20	18	17	12	12
Oxford Scale (0–6) Overall QOL			90	90	89	91	92	91	90	91	90	90	90	90
Disease activity:														
Brain (0–100)			2	2	**0**	0	0	0	0	0	0	0	0	0
Ears (0–100)			0	0	**0**	0	0	0	0	0	0	0	0	0
Eyes (0–100)			8	0	**0**	0	0	0	0	0	0	0	0	0

*ADL* Activities of daily living, *QOL* Quality of life

**Table 3:** Serial SuSx Scores

	Year 1	Week:	25	26	29	32	33	34	35	36	38	41	47	52
Patient 1														
*Susac symptoms scores:*														
Neurologic subscore														
Mean score (0–100)			1.1	0.3	2.5	1.6	2.5	2.7	2.3	2.9	3	2	1.8	1.8
Sub total			15	4	32	21	33	35	30	38	39	26	24	24
Ear subscore														
Mean score (0–100)			0	0	0	0	0	0	0	0	0	0	0	0
Sub total			0	0	0	0	0	0	0	0	0	0	0	0
Eye subscore														
Mean Score (0–100)			0	0	0	0	0	0	0	0	0	0	0	0
Sub total			0	0	0	0	0	0	0	0	0	0	0	0
Total symptoms score			15	4	32	21	33	35	30	38	39	26	24	24
Difficulty performing ADL (0–100)			0	**0**	0	0	0	0	0	0	0	0	0	0
Difficulty performing job (0–100)			6	6	12	10	15	15	15	15	15	15	13	12
Diminished QOL (0–100)			10	8	15	15	15	15	13	13	12	12	12	12
Total score for above 3			16	14	27	25	30	30	28	28	27	27	25	24
Oxford scale (0–6)					3	3	3	3	3	3	3	3	3	3
Overall QOL			91	91	89	75	75	75	78	79	79	80	80	80
Disease activity:														
Brain (0–100)			0	0	0	0	?	?	?	?	0	?	?	?
Ears (0–100)			0	0	0	0	0	0	0	0	0	0	0	0
Eyes (0–100)			0	0	0	0	0	0	0	0	0	0	0	0

*ADL* Activities of daily living, *QOL* Quality of life

**Table 4:** Serial SuSx Scores

	Year 2	Week:	11	27	47
Patient 1					
*Susac symptoms scores:*					
Neurologic subscore					
Mean score (0–100)			0.6	0.2	0.3
Sub total			8	2	4
Ear subscore					
Mean score (0–100)			0	0	0
Sub total			0	0	0
Eye subscore					
Mean score (0–100)			0	0	0
Sub total			0	0	0
Total symptoms score			8	2	4
Difficulty performing ADL (0–100)			0	0	0
Difficulty performing job (0–100)			5	3	5
Diminished QOL (0–100)			4	4	4
Total score for above 3			9	7	9
Oxford Scale (0–6)			2	2	2
Overall QOL			90	87	89
Disease activity:					
Brain (0–100)			0	0	0
Ears (0–100)			0	0	0
Eyes (0–100)			0	0	0

*ADL* Activities of daily living, *QOL* Quality of life

**Table 5:** Susac symptoms scores

	Patient 1		Patient 2	
	At Peak^1^	At 26 wks	At Peak^2^	At 27 wks
Neurologic symptoms subscore (0–1300)*:	898	4	810**	235
Decreased mental alertness (0–100)	62	0	80	0
Headache (0–100)**	51	0	**	0
Memory impairment (0–100)	96	0	85	25
Confusion/odd behavior (0–100)	61	0	80	25
Decreased executive function (0–100)	100	0	100	60
Personality change (0–100)	85	0	90	50
Emotional lability (0–100)	75	0	85	20
Intellectual impairment				
Affecting school/work (0–100)	100	4	100	55
Paresthesias (0–100)**	74	0	**	0
Imbalance/ataxia (0–100)	52	0	85	0
Difficulty walking (0–100)	44	0	80	0
Bladder dysfunction (0–100)	49	0	25	0
Apraxia (0–100)**	49	0	**	0
Inner ear symptoms subscore (0–500):	49	0	0	90
Hearing loss on R (0–100)	0	0	0	40
Hearing loss on L (0–100)	0	0	0	50
Tinnitus R (0–100)	0	0	0	0
Tinnitus L (0–100)	0	0	0	0
Vertigo (0–100)	49	0	0	0
Eye symptoms subscore (0–400):	0	0	0	0
Visual disturbance on R (0–100)	0	0	0	0
Visual disturbance on L (0–100)	0	0	0	0
Visual field loss on R (0–100)	0	0	0	
Visual field loss on L (0–100)	0	0	0	0
Total symptoms score (0–2200):	947	4	810**	325
Global assessment of disease activity in:				
Brain (0–100)	71	0	100	0
Inner ear (0–100)	33	0	0	0
Eyes (0–100)	0	0	0	0

^*^The second number in all parentheses represents the worst possible score

^**^Since some items (those with **) could not be scored, the subtotal is at least as high as shown

^1^One day before onset of treatment. ^2^Five weeks after onset of treatment

**Table 6:** SuS disease damage score at last visit

	Patient 1	Patient 2
Neurologic damage subscore (0–1700)*:	12 (13)**	325
Decreased mental alertness (0–100)	2 (2)	20
Slow thought processing (0–100)	2 (3)	10
Memory impairment (0–100)	0 (0)	20
Intellectual impairment		
Affecting school/work (0–100)	4 (3)	50
Decreased executive function (0–100)	0 (0)	65
Emotional lability (0–100)	0 (0)	30
Personality change (0–100)	0 (0)	25
Confusion/odd behavior (0–100)	0 (0)	25
Poor concentration (0–100)	4 (5)	50
Unsteady gait (0–100)	0 (0)	0
Spasticity (0–100)	0 (0)	0
Gross motor impairment (0–100)	0 (0)	10
Fine motor impairment (0–100)	0 (0)	10
Hemiparesis (0–100)	0 (0)	10
Neurogenic bladder (0–100)	0 (0)	0
Neurogenic bowel (0–100)	0 (0)	0
Slurred speech (0–100)	0 (0)	0
Inner ear damage subscore (0–600):	0 (0)	120
Hearing loss (H/L) R (0–100)	0 (0)	40
Hearing loss L (0–100)	0 (0)	50
Tinnitus R (0–100)	0 (0)	0
Tinnitus L (0–100)	0 (0)	0
Vertigo (0–100)	0 (0)	0
H/L adversely affecting QOL (0–100)	0 (0)	30
Eye damage subscore (0–500):	0 (0)	0
Permanent blind spot R (0–100)	0 (0)	0
Permanent blind spot L (0–100)	0 (0)	0
Constricted peripheral vision R (0–100)	0 (0)	0
Constricted peripheral vision L (0–100)	0 (0)	0
Visual damage affecting QOL	0 (0)	0
Total disease damage score (0–2800):	12 (13)	445
Global assessment of disease damage (0–100):	2 (5)	60

Last visit was at 23 months for case 1 and 21 months for case 2

^*^The second number in all parentheses represents the worst possible score

^**^ The attending physician’s scores for patient 1 are in parentheses

Figures 2 and 3 summarize immunosuppressive treatment during years 1 and 2. Tables 1, 2, 3, 4 document serial Susac Symptoms Scores from the onset of treatment to last follow-up visit 23 months later. These scores were generated by the family (the patient and her parents). Table 5 presents the data entered on the SuSx Form at two different points in time—the day before onset of treatment (at peak disease severity) and 26 weeks later—documenting marked improvement. Table 6 documents the patient’s DDS at her last visit, as judged by the family. This was corroborated by the patient’s attending physician (RTS).

### Patient 2

**Fig. 6:**
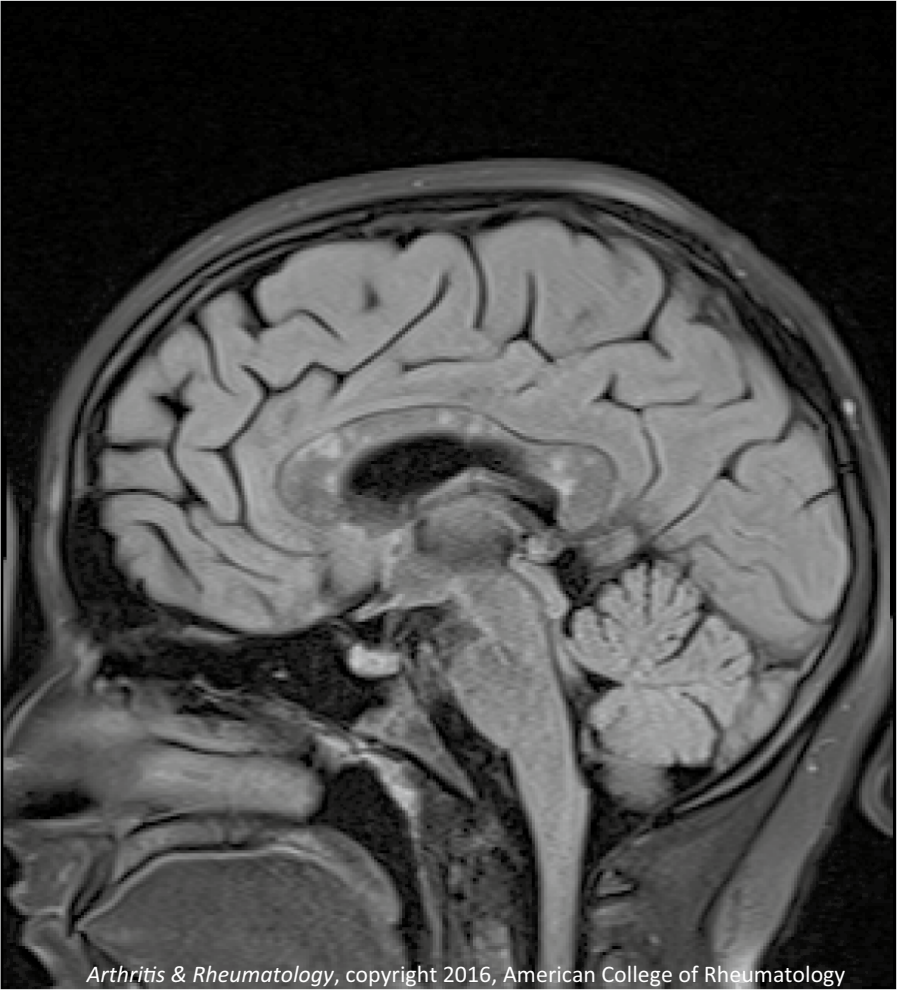
Corpus callosal lesions, case 2

**Fig. 7:**
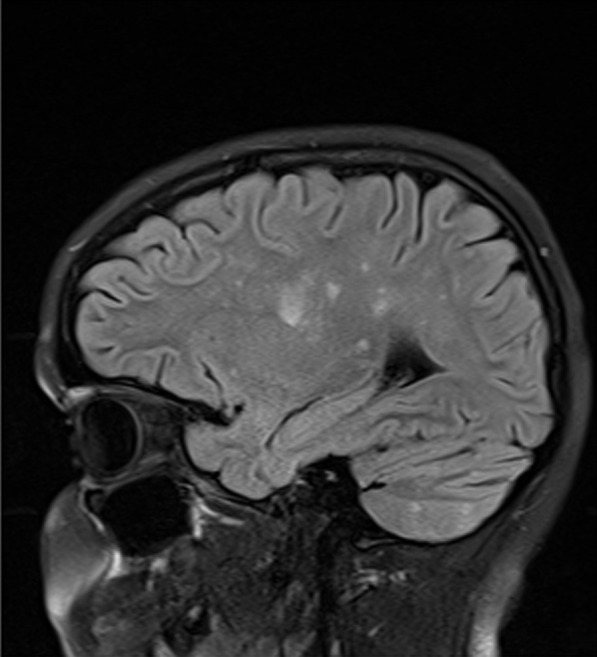
Extra-callosal lesions, case 2

**Fig. 8:**
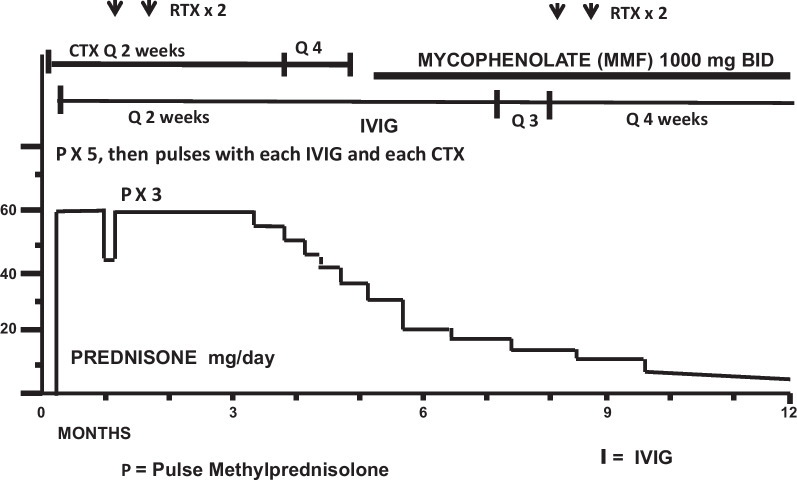
Immunosuppressive treatment: case 2, year 1

**Fig. 9:**
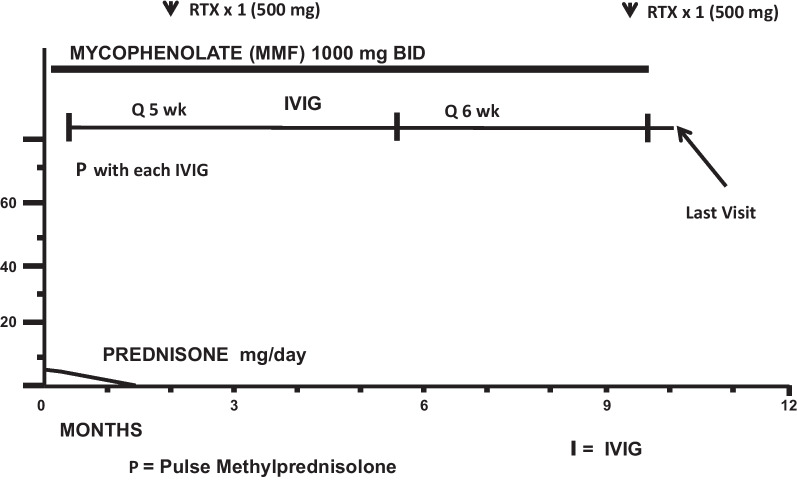
Immunosuppressive treatment: case 2, year 2

The second patient is a 14-year-old Caucasian female. She is the same patient who was briefly presented in an earlier publication [12]. She was in her usual state of health until she developed headache. On day 2 of her illness, she developed “slowed thinking” and difficulty verbalizing her thoughts. She intermittently “dazed off” and looked “glassy-eyed,” “not acting herself.” On day 4 she developed urinary incontinence. Over the next week these symptoms continued, and she additionally developed ataxia, fatigue, excessive sleeping, emotional lability, occasional vomiting, right hand numbness, and left jaw/lateral neck region pain. On day 12 she was admitted for intensive evaluation. Admission MRI (Figs. 6, 7) revealed multifocal T1 hypointensity and T2 hyperintensity lesions in the periventricular and supraventricular cerebral and cerebellar white matter, including extensive involvement of the corpus callosum. Cerebral spinal fluid (CSF) protein was elevated, with CSF white blood cell (WBC) of 5; erythrocyte sedimentation rate (ESR) was normal. On day 13, ophthalmological evaluation revealed evidence of branch retinal artery occlusion (BRAO) in the left eye. On day 15, she was started on aggressive immunosuppression. Details of her treatment, initially and during 21 months of follow-up, are shown in Figs. 8 and 9. Her weight and body surface area (BSA) at the onset of treatment were 84 kg and 1.92 M^2^, respectively.

**Fig. 10:**
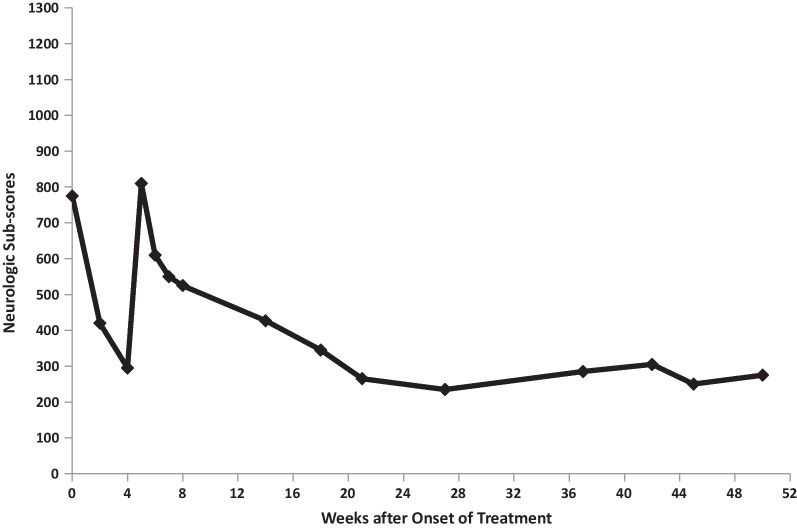
Serial neurologic subscores: case 2

**Fig. 11:**
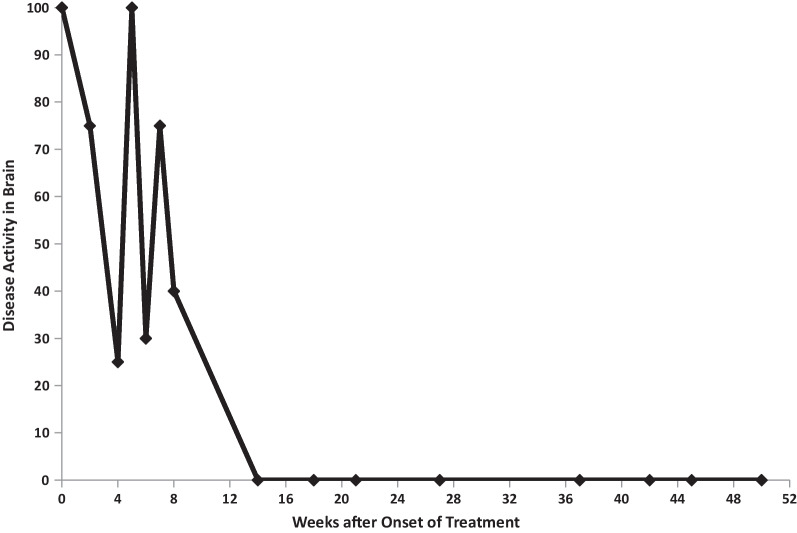
Serial disease activity scores: case 2

Her clinical course over the 21 months of her follow-up are documented by serial SuSx scores (Figs. 10, 11 and Tables 7, 8). Her SuSx Forms were completed by the physician who was most familiar with her (DRB) and were based on detailed narrative notes that had been prospectively entered into the patient’s medical records. Table 5 presents the data entered on the SuSx Form at two different points in time—the day before onset of treatment (at peak disease severity) and 27 weeks later.

**Table 7:** Serial SuSx Scores

Year 1	Week:	0	2	4	5	6	7	8	14	17	21	27	37	42
Patient 2														
Susac symptoms scores:														
Neurologic subscore														
Mean score (0–100)**		64.6	32.3	22.7	81.0	46.9	42.3	40.4	32.8	26.5	20.4	18.1	21.2	23.5
Sub total		775*	420	295	810*	610	550	525	427	345	265	235	285	305
Ear subscore														
score (0–100)		0	0	0	0	0	20	32	18	18	18	18	18	18
Sub total		0	0	0	0	0	100	160	90	90	90	90	90	90
Eye subscore														
Mean score (0–100)**		0	0	0	0	0	0	0	0	0	0	0	0	0
Sub total		0	0	0	0	0	0	0	0	0	0	0	0	0
Total symptoms score		775*	420	295	810*	610	650	685	517	435	355	325	375	395
Difficulty performing ADL (0–100)		80	25	0	90	10	60	60	20	10	5	5	5	10
Difficulty performing job (0–100)		100	90	80	100	95	90	90	60	65	50	50	50	65
Diminished QOL (0–100)		100	65	40	100	90	85	80	65	50	50	40	40	50
Total score for above 3		280	180	120	290	195	235	230	145	125	105	95	95	125
Oxford Scale (0–6)		5	5	4	6	5	5	5	5	5	5	5	5	5
Overall QOL (0–100)		0	30	60	0	20	15	20	35	45	50	60	60	50
Disease activity:														
Brain (0–100)		100	75	25	100	30	75	40	0	0	0	0	0	0
Ears (0–100)		0	0	0	0	0	50	40	0	0	0	0	0	0
Eyes (0–100)		0	0	0	0	0	0	0	0	0	0	0	0	0

*Week* Weeks after onset of treatment, *ADL* Activities of daily living, *QOL* Quality of life

*Means that one or more items could not be scored; so the subtotal is at least as high as shown

**Represents the mean score for those items that could be scored

**Table 8:** Serial SuSx Scores

	Year 1:	Week:	45	50	Year 2:	Week:	8	18	29	38
Patient 2										
Susac symptoms scores:										
Neurologic subscore										
Mean score (0–100)			19.2	21.2			18.8	17.7	15.4	19.6
Sub total			250	275			245	230	200	255
Ear subscore										
Mean score (0–100)			18	18			18	18	8	16
Sub total			90	90			90	90	40	80
Eye subscore										
Mean score (0–100)			0	0			0	0	0	0
Sub total			0	0			0	0	0	0
Total symptoms score			340	365			335	320	240	335
Difficulty performing ADL(0–100)			10	10			5	5	10	10
Difficulty performing Job(0–100)			60	60			60	60	50	70
Diminished QOL(0–100)			50	50			50	50	40	50
Total score for above 3			120	120			115	115	100	130
Oxford scale (0–6)			5	5			5	5	4	4
Overall QOL			50	50			50	60	65	60
Disease activity:										
Brain (0–100)			0	0			0	0	0	0
Ears (0–100)			0	0			0	0	0	0
Eyes (0–100)			0	0			0	0	0	0

*ADL* Activities of daily living, *QOL* Quality of life

After commencement of her immunosuppression, she initially improved, but then relapsed at the 5-week mark. This relapse was acute and intense. Her overall status became worse than at any time in the past: ataxia and urinary incontinence returned, she lost the ability to walk or even stand, she developed upper and lower extremity weakness, she became wheelchair bound, was unable to talk, became very emotionally labile, and for the first time, she developed hearing loss (moderate-severe, bilateral, from which she has never recovered).

A repeat MRI revealed multiple new diffusely scattered lesions, with restricted diffusion and enhancement. New diffuse leptomeningeal enhancement was also noted. Additional lesions were noted throughout the basal ganglia, thalami, putamen, and anterior limb of the internal capsule. Her serial SuSx scores (as presented in Table 7) documented the severity of this relapse. Over the course of 1 week, her neurological subscore rose from 295 to 810 (0 being normal, 1300 being the worst possible score) and her activities of daily living (ADL) score went from 0 to 90 (zero being normal and 100 being the worst possible score). Her neurologic subscore at week 5 (810) was worse than her neurologic subscore at the time of presentation (775). This acute relapse at the 5-week mark prompted escalation of immunosuppressive treatment. Her neurologic status slowly but steadily improved during weeks 6–21, but then did not significantly improve thereafter (Tables 7, 8). Since week 21, she has continued to have neurological deficits, apparently due to damage sustained during the first 5–6 weeks of her disease. This conclusion was drawn because disease activity in her brain (Fig. 11 and Table 7) seemed to either cease or become fully suppressed with treatment, and her persistently abnormal neurologic subscores did not worsen during considerable tapering of her immunosuppression. Subsequent to the week 5 resurgence, she experienced no flare-ups of disease activity in the brain, retina, or inner ear from week 14 through the rest of her 21 months of follow-up.

At her last visit, she was still experiencing considerable neurologic and cochlear symptomatology, all of which appeared to be due to disease damage, not to ongoing active disease.

In summary, this patient experienced a monocyclic course of SuS, which was characterized by extraordinarily intense/severe encephalopathy during the first 2–3 months of her disease. Her initial encephalopathy was difficult to control, despite prompt and aggressive immunosuppression. In fact, her disease surged severely 5 weeks after onset of treatment, despite her having received three pulses of cyclophosphamide. After 3 months of sustained, aggressive immunosuppression, her active disease finally subsided, such that by 14 weeks her disease appeared to be either inactive or fully suppressed. No further relapse has been apparent; however, during the time of her initial severe and unrelenting encephalopathy, she appears to have sustained significant damage despite aggressive treatment.

## Discussion

The Susac Symptoms (SuSx) Form was developed to help patients and their physicians more easily, accurately, and uniformly document the patient’s trajectory, with an ultimate goal of serially using the Form to guide real-time therapeutic decision-making for individual patients, while simultaneously generating data for research purposes. The DDS is intended to be used less frequently than the SuSx Form and informs interpretation of SuSx scores, while also serving as a long-term outcome measure. In an effort to achieve a balance of the forms being simple and practical yet also sufficiently complex and comprehensive to accurately capture clinical information, several patients with SuS participated in the design, trial, and ultimate decision-making regarding the final forms.

The data generated by the family of patient 1 demonstrate the value of prospectively using the SuSx Form, starting as soon as possible after diagnosis. Early in her disease course, we were prepared to quickly switch from mycophenolate mofetil (MMF) to cyclophosphamide if she did not adequately improve. Her serial scores documented sufficient improvement to allow us to continue MMF, and subsequent scores provided ongoing support for that decision and eventual tapering of her immunosuppression.

The data generated by patient 1 also demonstrate the value of empowering patients/families to play a major role in serial disease assessment. The assessments of the patient/family squared with and enhanced the clinical assessments and impressions of the physicians involved in her care. The family commented that the very process of prospectively completing the forms was therapeutic for them. It gave them an added sense of control, understanding, and reassurance regarding therapeutic decisions.

In the case of patient 2, the data were generated by the physician by retrospectively completing forms at key points along the patient’s clinical course. Though not prospectively completed, the data entered on the forms were based on careful, detailed narrative notes that had been prospectively kept. These data are still able to demonstrate the reason for certain treatment decisions and the patient’s longer-term outcome.

Although the SuSx Form was primarily designed to facilitate and improve therapeutic decision-making for individual patients, it was also designed to simultaneously generate data for clinical research purposes. From a research standpoint, the data generated by the two patients contribute several valuable observations. For example, the data from patient 1 document that a patient who presents with worrisome encephalopathy can have an excellent outcome when treated with MMF, rather than cyclophosphamide. The data from patient 2 demonstrate that it is sometimes very difficult to sufficiently control active Susac encephalopathy when it is initially extremely severe and unrelenting; that a surge of disease activity can occur even in the midst of treatment with cyclophosphamide; and that irreversible damage can occur despite early aggressive immunosuppression.

Although the data generated by the SuSx Form and the DDS appear to be valuable clinically and for research purposes, there are several limitations. Neither form has been statistically validated. Patients and families may vary considerably in the reliability of their data. Patient 1 and her parents fully grasped the concepts, fully studied and understood the Definitions and Gradations documents, showed excellent clinical judgment, and were highly committed to providing high-quality data. Other patients and families may not have the time or resources to achieve such reliability. It is essential to carefully coach patients before they complete their first Form. It is also important to review and critique their first completed Form and discuss and correct any misunderstandings regarding how to optimally complete the Form.

Although the SuSx Form is designed to provide information about disease activity, its scores do not necessarily reflect disease activity only—its scores may also reflect disease damage or temporary reversible organ injury. Accurate interpretation of whether a given symptom is due to active disease, incompletely healed organ injury with potential for at least some recovery, irreversible organ damage, or a mixture of these possibilities requires comparison of serial scores, and even then, clinical judgment is needed. Moreover, some of the symptoms may be due, in part, to factors other than Susac disease.

Because of the above factors, there are limitations regarding the extent to which one patient’s data should be compared with another patient’s data. For example, two patients who have identical SuSx neurologic subscores at the time of diagnosis may or may not truly have disease of equal severity. The main strength, then, is using scoring tendencies of individual patients/families to compare their recent scores with their past scores. Another limitation is that the value of the data declines if the forms are not completed with sufficient frequency, particularly during the most important early weeks and months of treatment. If forms are not completed at times of relapse, or at times of marked improvement, important fluctuations in disease activity may not be “captured” and the clinical course depicted by the serial data may be misleading.

Despite their limitations, the SuSx Form and the DDS provide useful data, particularly when used prospectively, but even when used retrospectively. Patient 1 and her parents have superbly demonstrated that patients/families can be empowered, enabled, encouraged, and enlisted to serve as thoughtful, competent patient–clinical researchers. The physician of patient 2 has demonstrated the value of sharing a careful retrospective reconstruction of an individual patient’s clinical course and response to treatment. The data suggest that these two Forms are practical and have potential to not only facilitate, expedite, and improve individual patient care, but also to uniformly collect much needed data on large numbers of patients for clinical research purposes. If such forms were to be completed prospectively and serially by many newly diagnosed patients, the data could add considerably to knowledge of the clinical courses, treatment needs, and outcomes of SuS. An important future step will be validation of the two forms.

## Conclusions

To date, most case reports on SuS have focused on clinical presentation and have contained a dearth of details regarding clinical course, course of treatment, and ultimate outcome. There has been a lack of uniformity in the reporting of cases. We would like to emphasize that future case reports on SuS could be of greater value, individually and collectively, if they include serial data generated by the SuSx form, outcome data generated by the DDS, and more details about treatment. In that sense, our case report is offered as a model for future case reporting on SuS, with the goal being to maximize the value of using case reports to further medical knowledge.

## Supplementary Information

**Additional file 1.** Susac Symptoms (SuSx) Form (Hand Version).

**Additional file 2.** Definitions and Gradations—for Susac Symptoms (SuSx) Form.

**Additional file 3.** Susac—Disease Damage Score.

**Additional file 4.** Definitions and Gradations—for DDS Form.

## Data Availability

Not applicable.

## Abbreviations

CSF: Cerebral spinal fluid
BRAO: Branch retinal artery occlusion
BSA: Body surface area
DDS: Disease damage score
DRB: Danielle R. Bullock
ESR: Erythrocyte sedimentation rate
FA: Fluorescein angiography
IV: Intravenous
IVIG: Intravenous immunoglobulin
JDM: Juvenile dermatomyositis
MMF: Mycophenolate mofetil
MRI: Magnetic resonance imaging
QOL: Quality of life
RTS: Robert T. Spencer
SuS: Susac syndrome
SuSx: Susac symptoms
WBC: White blood cell

## References

[1] Dorr J, Krautwald S, Wildemann B (2013). Characteristics of Susac syndrome: a review of all reported cases. Nat Rev Neurol.

[2] Kleffner I, Dunning T, Lohmann H (2012). A brief review of Susac syndrome. J Neurol Sci.

[3] Rennebohm R, Asdaghi N, Srivastava S, Gertner E (2018). Guidelines for the treatment of Susac syndrome. An update. Int J Stroke.

[4] Seifert-Held T (2017). Susac's syndrome: clinical course and epidemiology in a central European population. Int J Neurosci.

[5] Wilf-Yarkoni A, Elkayam O, Aizenstein O (2020). Increased incidence of Susac syndrome: a case series study. BMC Neurol.

[6] Gross CC, Meyer C, Bhatia U (2019). CD8^+^ T cell-mediated endotheliopathy is a targetable mechanism of neuro-inflammation in Susac syndrome. Nat Commun.

[7] Agamanolis DP, Klonk C, Bigley K (2019). Neuropathological findings in Susac syndrome: an autopsy report. J Neuropathol Exp Neurol.

[8] Agamanolis DP, Prayson RA, Asdaghi N (2019). Brain microvascular pathology in Susac syndrome—an electron microscopy study of 5 cases. Ultrastruct Pathol.

[9] Lei Y, Zhang J, Cara R, Schiavon CR (2021). SARS-CoV-2 spike protein impairs endothelial function via downregulation of ACE 2. Circ Res.

[10] Venditti L, Rousseau A, Ancelet C (2021). Susac syndrome following COVID-19 infection. Acta Neurol Belg.

[11] https://www.globalresearch.ca/19916-eye-disorders-including-blindness-following-covid-vaccine-reported-europe/5744219.

[12] Bullock DR, Rivera S, Beardsley RM (2016). Teen with encephalopathy: early recognition of and intervention for Susac Syndrome. Arthritis Rheumatol.

