# Supplementary material for: Use of disease assessment tools to increase the value of case reports on Susac syndrome: two case reports
Source: J Med Case Rep. 2023 Apr 13;17:158. doi: 10.1186/s13256-023-03838-9 (PMC10097450; doi:10.1186/s13256-023-03838-9)
Supplement: Supplementary file 1 — Additional file 1. Susac Symptoms (SuSx) Form (Hand Version). [file 13256_2023_3838_MOESM1_ESM.doc]

**Susac Symptoms (SuSx) Form (Hand Version)**

| **Patient ID#** |  | | (Month) (Day) (Year) |
| --- | --- | --- | --- |
| **This form was completed by:** | | ❒ Physician ❒Patient ❒ Family Member | |

**Instructions**:

- The Susac Symptoms form, which is the most important form of the ICS (especially for prospective use), is designed for both clinical care and research purposes. It may be completed by the physician, the patient, a family member, or each of these three---to indicate their opinions regarding the patient’s status on the date entered above. If the form is completed by a joint effort between the patient and family, you may check both Patient and Family. The form may be completed either prospectively or retrospectively (see last page).
- This form can be filled out much more easily on-line, using the REDCap system. This “Hand Version” is provided for those who would like to use a hand written version of the form, for their own purposes, in addition to the REDCap version.
- Please strike a short vertical line somewhere along the **horizontal line** (see examples below) to indicate the degree to which you think the patient has been experiencing each listed **symptom/abnormality** of Susac Syndrome **over the past week.** (Indicate a score that would be most representative for this past week.)
- When this form is printed out, each horizontal line is **10 cm (100 mm) long**. So, the degree to which a patient is experiencing the symptom could range from 0 (not experiencing the symptom at all) to 100 (experiencing the symptom to an extremely severe degree). Or, thinking in terms of “points,” 0 means the patient is experiencing 0 points of that particular symptom. 100 means the patient is experiencing 100 points of that symptom.
- To score a particular symptom/abnormality: **measure the distance (in millimeters) from the far left post (None, or 0) to where you have struck your vertical line**. Enter that numerical measurement in the rectangular box at the far right, as shown in the examples.
- For definitions of each symptom/abnormality, and for examples of various degrees of each, please refer to “**Susac Definitions and Gradations**” (which is a separate document). Use the Mild, Moderate, Severe, and Extremely Severe posts for guidance.
- If you think the patient has not been experiencing the symptom/abnormality at all, strike your line at the guidepost at the very farthest end on the left (at **None, or 0 points of that symptom**). See Example 1, below.
- If you think the patient has been experiencing the symptom/abnormality to a very extreme degree, strike your line at the guidepost at the very farthest end on the right (at **Extremely Severe, or 100 points of that symptom**). See Example 2.
- If you think the patient has experienced the symptom/abnormality to a degree somewhere between these two extremes, strike your line wherever seems most accurate. For example, if you think the patient has been experiencing the symptom to a mild-moderate degree, place your line somewhere between the Mild and Moderate guideposts (closer to Mild if that is the case; closer to Moderate if that is the case). See Example 3.

**None Mild Moderate Severe Extremely Severe**

**0 points 100 points**

**Example 1**

**0**

**100**

**Example 2**

**Example 3**

**32**

- When you strike your vertical line, you do **not** need to distinguish between abnormality that is due to active disease; abnormality that is due to permanent damage; abnormality due to slow recovery from temporary injury from past active (but no longer active) disease; abnormality due to your treatment; abnormality that is unrelated to your Susac syndrome; or abnormality that represents a mixture of these possible contributing causes. **Just** **indicate how much of the abnormality (symptom) is present, regardless of the cause(s)**.
- If the patient is extremely neurologically impaired (e.g. extremely listless, or almost comatose, or otherwise non-communicative) it may be impossible to assess the degree to which some of the 13 neurological symptoms are present. In such cases, place an **NA** (meaning **Not Assessable**) in the square box next to such a symptom and do not strike a vertical line along the horizontal line for that particular symptom.

| **Patient ID#** |  | **Date:**  (Month) (Day) (Year) |
| --- | --- | --- |

|  | | **None** | **Mild** | **Moderate** | **Severe** | **Extreme** |
| --- | --- | --- | --- | --- | --- | --- |
|  | | **0 100** | | | | |
| **Neurologic Symptoms (Abnormalities)** (Refer To Definitions And Gradations) | | | | | | |
| **Decreased Mental Alertness** | |  | | | | |
| **(slowed thinking, or worse)**  **Headache**  **Memory Impairment**  **Confusion or Odd**  **Behavior**  **Decreased Executive**  **Function**  **Personality Change**  **Emotional Lability**  **Intellectual Impairment**  **Affecting Work or School**  **Parasthesias (Numbness**  **Tingling)**  **Imbalance, Unsteadiness**  **of Gait, Ataxia (separate**  **from vertigo)**  **Difficulty Walking**    **Bladder Dysfunction**  **Apraxia**  **(Loss of ability to carry out a previously learned task, despite having the desire and physical ability to perform that task)** | |  | | | | |
|  | | **Neurologic Symptoms Subtotal (for the symptoms assessed):**  **Average Score for the Assessable Neurological Symptoms:**  **(The above Subtotal divided by 13)** | | | | |
|  | |  | | | | |
|  | |  | | | | |
|  | |  | | | | |
| **ID#** | | **Date:** | | | | |
|  | |  | | | | |
|  | |  | | | | |
|  | | | | | | |
| **Inner Ear Symptoms: None Mild Moderate Severe Extreme** | | | | | | |
| **Hearing loss on R** | | **0** **100** | | | | |
| **Hearing loss on L** | |  | | | | |
| **Tinnitus on R** | |  | | | | |
| **Tinnitus on L**  **Vertigo/Dizziness** | |  | | | | |
|  | |  | | | | |
| **Inner Ear Subtotal**    **Average Ear Score (the above 5 scores divided by 5)** | | | | | | |
| **Eyes Symptoms:** | | | | | | |
| **Visual Disturbance on R**  (How much visual disturbance, of any kind, have you typically noticed during this past week?)  **Visual Disturbance on L**  (How much visual disturbance, of any kind, have you typically noticed during this past week?)  **Visual Field Loss on R**  (What % of a normal visual field are you currently missing---that you have noticed?)  **Visual Field Loss on L**  (What % of a normal visual field are you currently missing---that you have noticed?) |  | | | | | |
|  | **Eye Subtotal**    **Average Eye Score (the above 4 scores divided by 4)** | | | | | |
| **Neurologic SubTotal Ear SubTotal Eye SubTotal**    **+ + = Total Symptom Score**  The maximum possible (worst possible) score is (13 x 100) + (5 x 100) + (4 x 100) = 2200 | | | | | | |
| **ID# Date:**    **Normal/None Mild Moderate Severe Extremely Abnormal**  **QUICK SUMMARY:**  **(See instructions on Page 6)**    **0 100**  **Difficulty Performing ADL**  **(Activities of Daily Living)**  **Difficulty Performing Job**  **Diminished Quality of Life**  **Total Score for Impaired Ability/Diminished QOL (total of the above 3)**  **Average Score for the above 3**  **Disease Activity Brain**  **Inner Ears**  **Eyes**  **Disease Damage Brain**  **Right Ear**    **Left Ear**  **Right Eye**  **Left Eye**  **Overall Quality of Life (QOL):**  **Oxford Handicap Score:**  Please place an X in the box next to the best description of your current situation. Choose only one. Your Oxford Handicap Score  is the number next to the box you have chosen. **Place that number in this box**  **1 NO SYMPTOMS**  **2** Minor symptoms, but **NOT INTERFERING WITH LIFESTYLE**.  **3** Minor handicap with symptoms leading to **SOME RESTRICTION IN LIFESTYLE**, but **NOT INTERFERING WITH THE PATIENT'S CAPACITY TO LOOK AFTER HIMSELF OR HERSELF**.  **4** Moderate handicap with symptoms that **SIGNIFICANTLY RESTRICT LIFESTYLE** and **PREVENT TOTALLY INDEPENDENT EXISTENCE**.  **5** Moderately severe handicap with symptoms that clearly **PREVENT INDEPENDENT EXISTENCE** though **NOT NEEDING CONSTANT ATTENTION**.  **6** Severe handicap leading to **TOTAL DEPENDENCE REQUIRING CONSTANT ATTENTION** during day and night. | | | | | | |

**Current Status of Visual Fields (Optional):**

Up

Far left periphery Nasal Far right periphery

Down

**Left Eye**  **Right Eye**

Please shade in any parts of your visual field in which vision is currently blocked or obscured. (Pretend that you are looking forward into the paper or computer screen.) We have not been able to enable you to fill this section out electronically. We suggest that you shade it in by hand; then either mail, fax, or scan-and-email it to us. If you wish, you may wait until you have accumulated several, then send.

**Further Description of Current Visual Symptoms:**

**Other Comments about How You are Currently Doing:**

**Current Medications:**

This section is optional (particularly if you are maintaining the Susac-Medication Flow Sheet), but is very helpful. Indicate (by placing an X in the box to the left of the listed medication) whether you are currently taking that medication. Then, enter the dose and frequency. For IVIG, enter the total number of grams given for that cycle. For example, if you are receiving 40 gm IVIG on Day 1 and 40 gm on Day 2 every 4 weeks, enter 80 gm (for the dose) and every 4 weeks (for the frequency). Do not list non-immunosuppressive medications.

**Prednisone Dose**

**Pulses of Methylprednisolone Dose Frequency**

**IVIG Dose Frequency Date Last Given (M/D/Y)**

**Mycophenolate Dose Cellcept? or Myfortic? Place an X in one of these 2 boxes**

**Cyclophosphamide Dose Frequency Date Last Given (M/D/Y)**

**Rituximab Dose Date Last Given (M/D/Y)**

**Other Immunosuppressive Medication(s); Other Comments/Clarifications:**

**ID # Date:**

**INSTRUCTIONS for the QUICK SUMMARY:**

For each item below, move the slider to the most appropriate point to indicate how much or how little problem the patient is currently having with that item---either on the exact date indicated at the beginning of this form, or on average during the week prior to that date, whichever is most representative.

**Difficulty Performing ADL (Activities of Daily Living):**

On a scale of 0 to 100---where 0= normal function (no difficulty) and 100= complete disability---to what extent has your (the patient's) Susac syndrome affected your (the patient's) ability to perform usual basic Activities of Daily Living (such as dressing, grooming, washing and drying your body, getting on and off the toilet, eating, preparing meals, getting in and out of bed or up from a chair, walking, climbing 5 steps, reaching for objects, bending to pick up things, doing normal chores, getting in and out of a car, running errands)? "Complete disability" means the patient is unable to perform any of these basic activities of daily living by him or herself. "Normal function" means that the patient is able to perform all of these basic activities independently and normally, without any difficulty, without any help from another person, and without help from an aid or adaptive device (cane, walker, etc.).

**Diffculty Performing Your Job:**

On a scale of 0 to 100---where 0= fully able to perform all of the work that you routinely performed in THE JOB YOU HAD AT THE TIME YOU BECAME ILL with Susac syndrome, and 100= unable to return to that job (because it is either impossible or would be unwise or irresponsible to do so)---to what extent do you (or would you) have difficulty performing that job. For young people who are in school (e.g. college), your job is being a student (studying and succeeding academically).

**Diminished Quality of Life (QOL):**

To what extent has your Susac syndrome by itself (everything associated with it) resulted in the current quality of your life being worse than it was before you developed Susac syndrome? 0= Not worse (i.e. your current QOL is the same or better than before Susac syndrome). 100= Your current QOL is extremely worse.

**Oxford Handicap Scale:**

Please indicate the description that best describes your current situation. Choose only one. Your Oxford Handicap Scale score (points of disability) is the number next to the description (box) you have chosen.

**Symptoms of Disease Activity:**

Scoring of this section is optional if you have been receiving treatment of your Susac syndrome for less than 6 months.

On a scale of 0-100, to what extent do you think you are having SYMPTOMS DUE TO INCOMPLETE SUPPRESSION OF ACTIVE DISEASE---in your brain, eyes, and ears? If you are having a symptoms, but you do not think the symptoms are due to incompletely suppressed active disease (and, instead, are entirely due to residual damage or slow recovery from past active disease, or you think the symptoms are due to emotional stress, medication side effects, or other non-Susac related factors), indicate zero.

On a scale of 0 to 100, 0= the patient is having no symptoms of active disease, either because the disease has become inactive (is in remission), or any active disease is being completely suppressed by current medications; and, any current symptoms are due either to residual damage from past active but no longer active disease, or to slow recovery from past active but no longer active disease, or are due to something other than incompletely suppressed active disease (like emotional stress or medication side effects). And, 100= an extreme amount of symptoms due to incompletely suppressed active disease---despite whatever immunosuppressive medication the patient may be receiving. If you think you are having minimal, mild, moderate, or severe symptoms due to minimal, mild, moderate, or severe amounts of unsuppressed disease activity, respectively, place the slider accordingly.

Again,"No symptoms of active disease" means that either your immune system has completely stopped making the Susac mistake (of attacking the microvasculature in your brain, for example) or your current medication is completely suppressing any active disease (such that there are no symptoms due to active disease) . Symptoms of "active disease" can occur if the current treatment is not fully suppressing the active disease, or if the active disease is not being treated at all. The amount of symptoms due to incompletely suppressed active disease can be minimal, mild, moderate, severe, or extremely severe.

**Disease Damage:**

Scoring of this section is optional if you have been receiving treatment of your Susac syndrome for less than 6 months.

On a scale of 0 to 100---where 0=no definite permanent damage has occurred, and 100=an extreme amount of definite, permanent damage has occurred---how much permanent damage do you think you have you sustained---in your Brain, Eyes, and Inner ears? Indicate damage only if you are quite certain that permanent damage has occurred. For example, if severe hearing loss has occurred and it has been concluded that this represents permanent severe hearing loss, indicate severe damage. But, if moderate hearing loss has only recently occurred and it is thought possible that it will return to normal, then do not indicate damage. Wait until it is clear that permanent damage has occurred.

**Overall Quality of Life:**

For this question, think of your life as a whole, in its entirety, not just the extent to which it has been affected by Susac syndrome. On a scale of 0 to 100---where 0=an extremely poor quality of life and 100= excellent (the very best quality of life that anyone could realistically and practically expect to have)---what is your current quality of life? Move the slider to the point that best indicates your quality of life. NOTE: For this particular question, unlike all of the other questions, A HIGH NUMBER IS GOOD.

**Please either Scan-and-Email, FAX, or mail to Dr. Rennebohm at:**

**FAX: 216 636 3002**

**Email:** [**rennebr@ccf.org**](mailto:rennebr@ccf.org)

**Robert Rennebohm, MD**

**Director, Susac Syndrome Consultation Services**

**Crile Bldg, Desk A111, Attn: Janica Petty**

**Cleveland Clinic**

**9500 Euclid Ave.**

**Cleveland, OH 44195**

**USA**

**Place an X in the first box below, if you have filled this form out retrospectively---to indicate the patient’s status on a date in the past. In such an instance, the date entered on all pages should be that past date.**

**If you have filled this form out retrospectively, place the current date here:**

**MM/DD/YY: ___ / ____ / ____**
