# Supplementary material for: Use of disease assessment tools to increase the value of case reports on Susac syndrome: two case reports
Source: J Med Case Rep. 2023 Apr 13;17:158. doi: 10.1186/s13256-023-03838-9 (PMC10097450; doi:10.1186/s13256-023-03838-9)
Supplement: Supplementary file 2 — Additional file 2. Definitions and Gradations—for Susac Symptoms (SuSx) Form. [file 13256_2023_3838_MOESM2_ESM.doc]

**Definitions and Gradations---for Susac Symptoms (SuSx) Form**

**Decreased Mental Alertness/Responsiveness:** To what extent does the patient’s mind seem fully alert and normally responsive, as opposed to dull, sluggish, slow, or listless?

- None: Normally alert; fully and normally responsive.
- Mild: A little sluggish, a little listless. Mind is not as alert as when was healthy, thought processing is a little slow, but patient is able to do all activities of daily living (ADL) within the home, without assistance. Not sufficiently alert to function adequately at school or work.
- Moderate: Obviously sluggish, slow, and listless. Sufficiently alert to ambulate, but only with assistance. Needs assistance with ADL.
- Severe: Stuporous. Opens eyes upon stimulation. Follows at least one step instructions, but has difficulty with much more. Bed-ridden because of the degree of listlessness.
- Extremely Severe: Comatose, unconscious, non-responsive. Unable to arouse, even when stimulated with deep pain.

**Headache:** To what extent does the patient have more frequent or more severe headaches than a healthy person?

- None: No headaches; or no different from healthy individuals.
- Mild: Has more frequent or more severe headaches than seems normal, but the headaches never, or only rarely, interfere with the patient’s usual activities of daily living.
- Moderate: The headaches significantly interfere with some activities, and more than just rarely.
- Severe: The headaches are daily and often incapacitating.
- Extremely Severe: Incapacitating and causing vomiting and hospitalization.

**Memory Impairment (Short term memory):** To what extent does the patient have difficulty with immediate recall and recent memory? (Do not consider past and very remote memory.)

- None: No impairment.
- Mild: A little worse than the average person’s memory would be. Almost never a significant problem, though.
- Moderate: More than a little worse, but not much worse. Sometimes a considerable problem.
- Severe: Much worse than the average person. Often a considerable problem.
- Extremely Severe: Unable to remember anything just told to him/her.

**Confusion or Odd Behavior:** To what extent is the patient (at least on occasion) exhibiting odd, unexpected, inappropriate behavior; seeming to be confused, disoriented, "out of it;" behaving uncharacteristically irresponsible, etc.?

- None: There has been nothing odd about his/her behavior. No confusion.
- Mild: Occasionally behaves in a somewhat odd or uncharacteristic way. Or, makes an occasional odd decision, but nothing dramatic and most people would not have noticed. Or, is slightly confused at times.
- Moderate: Sometimes behaves oddly; more than a little odd; but not dramatically odd. Or, is definitely confused at times, but not severely so.
- Severe: Often makes very poor and dangerous decisions and/or exhibits highly unusual or greatly confused behavior.
- Extremely Severe: Exhibits “shockingly” odd, or extremely confused, or extremely embarrassing and irresponsible behavior.

**Decreased Executive Function:** To what extent, currently, are the patient’s executive capabilities diminished, compared to his/her capacities prior to developing Susac Syndrome? “Executive function” refers to a person’s capacity to perform the tasks of executives and leaders (or the tasks of mothers and fathers). This includes decision making, organizing, problem solving, complex planning, making difficult judgments, showing leadership, and responsibly looking after children.

- None: Not diminished at all.
- Mild: Slightly diminished, but people would probably not notice this diminished capacity.
- Moderate: More than slight, but not severe.
- Severe: Severely diminished. Would have difficulty that would be obvious to everyone. Could handle only the simplest of executive functions.
- Extremely Severe: Patient is completely unable to carry out any executive functions. It would be inappropriate to even ask him/her to try.

**Personality Change:** To what extent has the patient’s personality changed? Personality, by definition, means the individual characteristics, qualities, tendencies, and temperament that make a person a distinct individual. Has the patient, for example, become more passive, more aggressive, less outgoing, more demanding, more docile, more irritable, more angry, or more apathetic?

- None: No change in personality or temperament.
- Mild: Has changed a little.
- Moderate: Has changed more than a little, but not a whole lot.
- Severe: Has changed a great deal.
- Extremely Severe: Patient’s personality has changed so much that he/she seems like a completely different person.

**Emotional Lability:** To what extent has the patient become excessively “emotional,” with excessively wide mood swings; with, for example, frequent inexplicable, inappropriate, or excessive crying or laughing?

- None: No emotional lability.
- Mild: Occasionally seems to cry or laugh inappropriately or excessively, but not dramatically so, and not every day.
- Moderate: Often cries or laughs inappropriately, inexplicably, or excessively and fairly dramatically so—but no more than once or twice per day.
- Severe: Very frequent and obvious episodes of crying, over which the patient seems to have absolutely no control. Happens several times per day.
- Extremely Severe: Almost constantly inexplicably crying or laughing.

**Intellectual Impairment Affecting Work/School:** To what extent is intellectual (cognitive) impairment currently making it difficult for the patient to function in normal expected fashion at school or in the workplace?

- None: Able to fully function at school or work, **as before illness.**
- Mild: Has slight impairment, but is able to compensate so that most people notice no impairment.
- Moderate: Has impairment that is obvious to co-workers/classmates, but is able to function if a few adaptations are made.
- Severe: Is able to do only some of the usual work expected and needs many special adaptations.
- Extremely Severe: Patient is unable to do any of the school work or work that he/she was able to do prior to this illness. Unable to be employed. Unable to do school work, even at home, even with a tutor.

**Paresthesias (Numbness,Tingling):** To what extent does the patient notice numbness or tingling in the extremities or in the face?

- None: No numbness or tingling anywhere.
- Mild: Any numbness or tingling is either mild, involves only a small area, or is only briefly and intermittently present.
- Moderate: Numbness and tingling that is worse than mild, but not severe.
- Severe: Numbness that is constantly present and at least moderately severe.
- Extremely severe: Numbness or tingling that is very severe, painful, and constantly present.

**Imbalance/Unsteadiness:** We are not talking about vertigo or dizziness here. We are talking about unsteadiness of gait, easy loss of balance (as in ataxia).

- None: Normal, steady gait.
- Mild: Mild or only occasional unsteadiness.
- Moderate: Moderate or frequent unsteadiness. May need to “hold onto walls” at times
- Severe: Very frequent and severe unsteadiness, or constant moderate unsteadiness.
- Extremely Severe: constantly has to hold onto walls; so unsteady that has to avoid walking.

**Difficulty Walking:** How much difficulty is the patient having with walking?

- None: Able to walk normally.
- Mild: Able to walk by self (without a walker or assistance from another person), but walks abnormally slowly and weakly.
- Moderate: Able to walk many steps, but only slowly and only with the help of a walker or an assisting person.
- Severe: Able to stand up (with or without assistance), but able to take only a few (2-4) steps and needs assistance from another person in order to take those few steps.
- Extremely Severe: Patient is bedridden. Unable to even stand-up by self, even with the support of an assisting person.

**Bladder Dysfunction**: Abnormal urinary bladder function caused by a problem with the nervous system. Spontaneous, uncontrolled nerve impulses to the bladder trigger spastic, unexpected bladder contractions, resulting in accidental voiding or excessively frequent need to urinate.

- None: Normal bladder function.
- Mild: Occasionally has abnormal bladder function, but just mild and intermittent.
- Moderate: Every day, the patient needs to urinate with abnormal frequency, but has no accidents.
- Severe: Patient constantly deals with urinary frequency and frequently has accidental voiding.
- Extremely Severe: Patient is unable to void on his/her own. Patient requires daily catheterization to evacuate the bladder.

**Apraxia:** Apraxia means loss of ability to carry out a previously learned and mastered task, despite having the desire and physical ability to perform that task. You know what physical task you want to do, and you know (or at least used to know) how to do it, but you cannot get the task done because the brain has difficulty getting the muscles to do what they need to do.

- None: No episodes of apraxia.
- Mild: Rare, brief episodes of mild apraxia. Not interfering with work.
- Moderate: Fairly frequent mild episodes, or more disturbing or alarming episodes.
- Severe: Frequent, disturbing, alarming episodes. Interfering with work competence.
- Extremely Severe: Very frequent, very alarming, incapacitating.

**______________________________________________________________________________**

**Hearing Loss:**

- None: Patient has not noted any hearing loss. Audiogram, if done, has been normal.
- Slight: Patient suspects slight hearing loss, slight muffling or distortion of sound; but an audiogram is normal.
- Mild: Mild hearing loss noted by patient and on audiogram.
- Moderate: Moderate hearing loss noted by patient and on audiogram. hearing aids have been suggested, at least as an appropriate option.
- Severe: Severe hearing loss, using hearing aid. Cochlear implant has been recommended, or at least offered as an option.
- Extremely Severe: Patient has undergone Cochlear implantation.

**Tinnitus:** Tinnitus means “ringing” in the ears. To what extent does the patient have tinnitus?

- None: No tinnitus.
- Mild: Patient has tinnitus, but is largely oblivious to it. Usually doesn’t notice it
- Moderate: Fairly bothersome tinnitus, but quite tolerable.
- Severe: Loud tinnitus. Barely tolerable.
- Extremely Severe: Has constant very loud, “roaring tinnitus” that is intolerable and “driving patient crazy.”

**Dizziness/Vertigo:** To what extent does the patient have a sensation of spinning or whirling—a sensation of objects rotating about the patient, or of the patient rotating about objects?

- None: No vertigo.
- Mild: Occasional mild vertigo, but able to function almost normally.
- Moderate: Moderate vertigo when upright. Functional only while in supine position.
- Severe: No vertigo while supine; severe vertigo when sits up or stands. Largely incapacitated.
- Extremely Severe: The whirling sensation is so severe and constant that the patient is constantly nauseated, frequently vomiting, and unable to assume anything other than a motionless supine position. Totally incapacitated by the vertigo.

**_____________________________________________________________________________________**

**Visual Disturbance:** Various kinds/types of visual disturbance may be noted by patients with Susac syndrome. The most severe type would be a fixed scotoma---which means that there is a portion of your visual field (in one eye) that is blocked by a dark spot (of various size and shape), as if a shade has been pulled over that part of your visual field. A scotoma can be “fixed” or “transient.” A “fixed scotoma” means that the dark spot is constantly present (possibly even for years), though it may eventually be noticed less. A “transient scotoma” is one that is present for only a brief period of time (minutes, hours, days, or weeks) and eventually disappears. Other visual disturbances are much more vague than scotoma---like intermittent “flashes,” intermittent brief images that dart into the peripheral vision, or intermittent distortions of vision.

- None: No visual disturbance noted.
- Mild: Occasional, mild, vague, ill-defined, difficult to describe visual disturbance, but not scotoma.
- Moderate: Frequent mild-moderate, ill-defined, difficult to describe visual disturbance, but not scotoma.
- Severe: Definite scotoma, but not fixed.
- Extremely Severe: Fixed scotoma.

**Visual Field Loss:** The purpose of this question is to document the size of any scotoma/scotomas (fixed or transient) that you are noticing---expressed as the percentage of your visual field that you are missing because of one or more scotomas. If you have no scotoma, then you are missing 0% of your visual field. If you have two scotoma in your right eye, one blocking 20% of your visual field in that eye and the other blocking 15%, then these two scotomas have caused you to lose 35% of your visual field in the right eye.

**___________________________________________________________________________________**

**Other Definitions: (For completion of the Susac-Cumulative Manifestations List and other Forms)**

**Spasticity:** Increased tone of muscles. For example, with spasticity of the legs there is an increase in the leg muscles’ tone so they feel tight and rigid and the knee jerk reflex is exaggerated.

- None: Normal muscle tone and reflexes.
- Mild: Extremity muscles are tight and reflexes are abnormally increased, but only the patient and physician are aware of it, and the spasticity does not interfere with physical function.
- Moderate: Patient’s spasticity is evident to those who watch the patient walk, but it is relatively subtle.
- Severe: Patient’s spasticity is very obvious to those who watch the patient walk, but patient is able to ambulate fairly easily and carry out most physical functions.
- Extremely Severe: Patient’s spasticity is very obvious and interferes greatly with physical function.

**Motor Apraxia**: Impaired ability to execute a motor function, because the brain has difficulty getting the muscles to do what they need to do.

**Difficulty speaking (expressive aphasia):** To what extent is it difficult for the patient to actually say words that are in his/her mind? For example, if the patient wants to say “Hello,” is he/she able to actually say that word out loud?

**Difficulty comprehending speech (auditory aphasia)**: To what extent does the patient have difficulty comprehending auditory forms of communication?

**Nominal Aphasia:** To what extent does the patient have difficulty naming persons or objects?

**Dark spots:** The patient sees “dark spots” or “black areas” (scotoma) in their visual field.

**Hemiparesis**: Weakness of an arm or leg; slight paralysis of an arm or leg.
