# Supplementary material for: Use of disease assessment tools to increase the value of case reports on Susac syndrome: two case reports
Source: J Med Case Rep. 2023 Apr 13;17:158. doi: 10.1186/s13256-023-03838-9 (PMC10097450; doi:10.1186/s13256-023-03838-9)
Supplement: Supplementary file 3 — Additional file 3. Susac—Disease Damage Score. [file 13256_2023_3838_MOESM3_ESM.doc]

**Susac---Disease Damage Score (Susac-DDS)**

| **Patient ID#** |  | | **Date**  (Mon) (Day) (Year) |
| --- | --- | --- | --- |
| **This form was completed by:** | | ❒ Physician ❒Patient ❒Family Member | |

**Instructions**:

This form is to be completed by the patient, or a family member, or the physician, or all three. Our preference is for patients to complete this form on-line, using the REDCap system, but this version of the form may be completed by hand.

The form is designed to capture the extent to which you think the patient's Susac syndrome has resulted in any permanent damage (irreversible harm) to the brain, eyes, or ears.

If your Susac syndrome has been treated for less than 6 months, you need not complete this form---because, during the first 6 months, it is usually too difficult, even impossible, to know whether a problem is due to permanent damage or not.

We realize that it is often impossible to know for sure whether a given problem is potentially reversible or surely permanent. We just want your best guess. It should be understood that symptoms or problems that are definitely due to permanent damage may improve, at least slightly, over time---because of practice, physical/occupational therapy, or compensation by the brain---or, may temporarily worsen during times of emotional stress, or when you are very tired.

As a general rule, a problem (e.g. moderately severe hearing loss) is probably due to permanent damage if it has not improved over the course of 6 months, or more, AND, if it has neither worsened when immunosuppressive treatment has been reduced, nor improved when immunosuppression has been increased. That is, the symptom stays the same, despite changes in treatment. The longer such a symptom remains unchanged like this, the more likely it is that the symptom is due to permanent damage.

**When this form is downloaded from our website and printed out**, each horizontal line is **10 cm (100 mm) long**. So, the degree to which a patient is experiencing the listed abnormality could range from 0 (not experiencing the abnormality at all) to 100 (experiencing the abnormality to an extremely severe degree). Or, thinking in terms of “points,” 0 means the patient is experiencing 0 points of that particular abnormality. 100 means the patient is experiencing 100 points of that abnormality.

There are 28 listed abnormalities (potential symptoms/signs of damage). **The worst possible damage score is 28 x 100=2800**, meaning that a score of 2800 would represent the worst possible amount of permanent damage, and a score of 0 would mean no permanent damage.

For definitions of the symptoms/problems, please refer to the document entitled "**Susac Definitions and Gradations (Susac-DG)**"

**For each listed symptom/problem, indicate the extent to which you think permanent damage (irreversible harm) of this type has occured---by striking a short vertical line somewhere along the 100 mm line**.

For example, if the patient has a symptom/problem (e.g. hearing loss, vision loss, or decreased mental alertness) and you think it probably or definitely represents a Moderate-Severe degree of permanent abnormality, strike your vertical line somewhere between the Moderate and Severe guideposts on the horizontal line. **The damage score for that item is the distance (in mm) from the far left end of the horizontal line (from None) to where you have struck your vertical line. In the Example, below, the distance is 65 mm---so, the score is 65.**

If the patient has a symptom (e.g. decreased mental alertness, or hearing loss, or vision loss), but you think it is all due to active disease (or slow recovery from temporary injury caused by past active, but no longer active disease), and you do not think it is permanent (or at least, you do not think you can declare it permanent, yet), then strike your vertical line all the way to the left, at "None."

Example:

|  | **None** | **Mild** | **Moderate** | **Severe** | **Extreme** |
| --- | --- | --- | --- | --- | --- |
|  | **0 100** | | | | |

**65**

**Hearing Loss**

| **Patient ID#** |  | **Date**  (Month) (Day) (Year) |
| --- | --- | --- |

|  | **None** | **Mild** | **Moderate** | **Severe** | **Extreme** |
| --- | --- | --- | --- | --- | --- |
|  | **0 100** | | | | |
| **Neurologic Manifestations:** | | | | | |
| Decreased Mental Alertness (Mental Sharpness) |  | | | | |
| Slow Thought Processing |  | | | | |
| Memory Impairment |  | | | | |
| Intellectual impairment affecting school/work |  | | | | |
| Decreased Executive Function |  | | | | |
| Emotional Lability |  | | | | |
| Personality Change |  | | | | |
| Confusion/Odd Behavior |  | | | | |
| Poor concentration/attention |  | | | | |
| Unsteady Gait |  | | | | |
| Spasticity |  | | | | |
| Gross Motor Impairment |  | | | | |
| Fine Motor Impairment |  | | | | |
| Hemiparesis |  | | | | |
| Neurogenic bladder |  | | | | |
| Neurogenic Bowel |  | | | | |
| Slurred Speech |  | | | | |
|  |  | | | | |
| **Neurologic Sub Total** | | | | | |

| **Patient ID#** |  | **Date**  (Mon) (Day) (Year) |
| --- | --- | --- |

|  | **None** | **Mild** | **Moderate** | **Severe** | **Extreme** |  |
| --- | --- | --- | --- | --- | --- | --- |
|  | **0 100** | | | | |  |
| **Inner Ear Damage :** | | | | | |  |
| Hearing Loss (H/L) on Right |  | | | | |  |
| Hearing Loss on Left |  | | | | |  |
| Tinnitus on R  Tinnitus on L |  | | | | |  |
| Dizziness/Vertigo  Extent to which H/L is Adversely Affecting QOL |  | | | | |  |
| **Inner Ear Sub Total** | | | | | |  |
| **Eye Damage:** | | | | | |  |
| Permanent blind spot(s) on R |  | | | | |  |
| Permanent blind spot(s) on L |  | | | | |  |
| Constricted peripheral vision on R |  | | | | |  |
| Constricted peripheral vision on L  Extent to which Visual Damage is Affecting QOL |  | | | | |  |
|  |  | | | | |  |
| **Eye Sub Total** | | | | | |  |
| **Comments, Regarding Eyes or Ears:** | | | | | |  |
|  |  | | | | |  |
|  |  | | | | |  |
|  |  | | | | |  |
|  |  | | | | |  |
| **Total Disease Damage Score (DDS)----Neurologic + Inner Ear + Eye Sub Totals** | | | | | |  |
|  | | | | | |  |

**Patient ID # ____________________________ Date _____ ______ ______**

(Mon) (Day) (Year)

**The Global Assessment of Disease Damage (GA-DD) (immediately below) is scored separately**

| **Global Assessment of Disease Damage (GA-DD)** | | | | | |
| --- | --- | --- | --- | --- | --- |
|  | **None** | **Mild** | **Moderate** | **Severe** | **Extreme** |
|  | **0 100** | | | | |
| Physician |  | | | | |
| Patient |  | | | | |
| Family |  | | | | |

**Instructions for the Global Assessment of Disease Damage (GA-DD):**

Strike a short vertical line somewhere along the 100 mm horizontal line to indicate your estimate of the **overall** amount and severity of damage that you think Susac syndrome has created for the patient. Consider all components of the disease---brain involvement, eye involvement, inner ear involvement, as a whole. Also, consider the extent to which the patient’ Susac syndrome, overall, has damaged the patient's quality of life.

The score for this section (the GA-DD Score) is the distance (measured in mm) from the far left end of the horizontal line to the point where you struck the vertical line. The maximum possible score is 100; the best possible score (no damage) is 0. Enter the measured distance (the score) into the box on the far right.

If you are the patient, use only the line next to Patient. If you are a family member, use only the line next to Family. If you are the physician, use only the line next to Physician.
