# Supplementary material for: Use of disease assessment tools to increase the value of case reports on Susac syndrome: two case reports
Source: J Med Case Rep. 2023 Apr 13;17:158. doi: 10.1186/s13256-023-03838-9 (PMC10097450; doi:10.1186/s13256-023-03838-9)
Supplement: Supplementary file 4 — Additional file 4. Definitions and Gradations—for DDS Form. [file 13256_2023_3838_MOESM4_ESM.doc]

**Definitions and Gradations---for DDS Form**

**Neurologic Harm:**

**Decreased Mental Alertness/Responsiveness:** To what extent does the patient’s mind seem fully alert and normally responsive, as opposed to dull, sluggish, slow, or listless?

- None: Normally alert; fully and normally responsive.
- Mild: A little sluggish, a little listless. Mind is not as alert as when was healthy, thought processing is a little slow, but patient is able to do all activities of daily living (ADL) within the home, without assistance. Not sufficiently alert to function adequately at school or work.
- Moderate: Obviously sluggish, slow, and listless. Sufficiently alert to ambulate, but only with assistance. Needs assistance with ADL.
- Severe: Stuporous. Opens eyes upon stimulation. Follows at least one step instructions, but has difficulty with much more. Bed-ridden because of the degree of listlessness.
- Extremely Severe: Comatose, unconscious, non-responsive. Unable to arouse, even when stimulated with deep pain.

**Slow Thought Processing:** This question is similar to the one above, but focuses more specifically on the extent to which the patient’s thought processing and problem-solving seems to be slower than it was prior to developing Susac syndrome:

- None: Not at all slow. Just as quick as it was before Susac syndrome.
- Mild: Has only slight or mild slowing of thought processing and difficulty with problem-solving
- Moderate: Able to process thoughts and solve problems, but only quite slowly and with considerable difficulty.
- Severe: Capable of processing only simple thoughts, and does so slowly.
- Extremely Severe: Unable to process any thoughts or solve even the simplest of problems.

**Memory Impairment (Short term memory):** To what extent does the patient have difficulty with immediate recall and recent memory? (Do not consider past and very remote memory.)

- None: No impairment.
- Mild: A little worse than the average person’s memory would be. Almost never a significant problem, though.
- Moderate: More than a little worse, but not much worse. Sometimes a considerable problem.
- Severe: Much worse than the average person. Often a considerable problem.
- Extremely Severe: Unable to remember anything just told to him/her.

**Intellectual Impairment Affecting Work/School:** To what extent is intellectual (cognitive) impairment currently making it difficult for the patient to function in normal expected fashion at school or in the workplace?

- None: Able to fully function at school or work, **as before illness.**
- Mild: Has slight impairment, but is able to compensate so that most people notice no impairment.
- Moderate: Has impairment that is obvious to co-workers/classmates, but is able to function if a few adaptations are made.
- Severe: Is able to do only some of the usual work expected and needs many special adaptations.
- Extremely Severe: Patient is unable to do any of the school work or work that he/she was able to do prior to this illness. Unable to be employed. Unable to do school work, even at home, even with a tutor.

**Decreased Executive Function:** To what extent, currently, are the patient’s executive capabilities diminished, compared to his/her capacities prior to developing Susac Syndrome? “Executive function” refers to a person’s capacity to perform the tasks of executives and leaders (or the tasks of mothers and fathers). This includes decision making, organizing, problem solving, complex planning, making difficult judgments, showing leadership, and responsibly looking after children.

- None: Not diminished at all.
- Mild: Slightly diminished, but people would probably not notice this diminished capacity.
- Moderate: More than slight, but not severe.
- Severe: Severely diminished. Would have difficulty that would be obvious to everyone. Could handle only the simplest of executive functions.
- Extremely Severe: Patient is completely unable to carry out any executive functions. It would be inappropriate to even ask him/her to try.

**Emotional Lability:** To what extent has the patient become excessively “emotional,” with excessively wide mood swings; with, for example, frequent inexplicable, inappropriate, or excessive crying or laughing?

- None: No emotional lability.
- Mild: Occasionally seems to cry or laugh inappropriately or excessively, but not dramatically so, and not every day.
- Moderate: Often cries or laughs inappropriately, inexplicably, or excessively and fairly dramatically so—but no more than once or twice per day.
- Severe: Very frequent and obvious episodes of crying, over which the patient seems to have absolutely no control. Happens several times per day.
- Extremely Severe: Almost constantly inexplicably crying or laughing.

**Personality Change:** To what extent has the patient’s personality changed? Personality, by definition, means the individual characteristics, qualities, tendencies, and temperament that make a person a distinct individual. Has the patient, for example, become more passive, more aggressive, less outgoing, more demanding, more docile, more irritable, more angry, or more apathetic?

- None: No change in personality or temperament.
- Mild: Has changed a little.
- Moderate: Has changed more than a little, but not a whole lot.
- Severe: Has changed a great deal.
- Extremely Severe: Patient’s personality has changed so much that he/she seems like a completely different person.

**Confusion or Odd Behavior:** To what extent is the patient (at least on occasion) exhibiting odd, unexpected, inappropriate behavior; seeming to be confused, disoriented, "out of it;" behaving uncharacteristically irresponsible, etc.?

- None: There has been nothing odd about his/her behavior. No confusion.
- Mild: Occasionally behaves in a somewhat odd or uncharacteristic way. Or, makes an occasional odd decision, but nothing dramatic and most people would not have noticed. Or, is slightly confused at times.
- Moderate: Sometimes behaves oddly; more than a little odd; but not dramatically odd. Or, is definitely confused at times, but not severely so.
- Severe: Often makes very poor and dangerous decisions and/or exhibits highly unusual or greatly confused behavior.
- Extremely Severe: Exhibits “shockingly” odd, or extremely confused, or extremely embarrassing and irresponsible behavior.

**Poor Concentration/Attention:**

- None: No difficulty concentrating or maintaining focus.
- Mild: Mild difficulty with concentration and attention span.
- Moderate: Moderate difficulty with concentration and attention span.
- Severe: Severely reduced attention span and ability to focus.
- Extremely Severe: Unable to focus or concentrate at all.

**Unsteady Gait:** We are not talking about vertigo or dizziness here. We are talking about unsteadiness of gait, easy loss of balance (as in ataxia).

- None: Normal, steady gait.
- Mild: Mild or only occasional unsteadiness.
- Moderate: Moderate or frequent unsteadiness. May need to “hold onto walls” at times
- Severe: Very frequent and severe unsteadiness, or constant moderate unsteadiness.
- Extremely Severe: constantly has to hold onto walls; so unsteady that has to avoid walking.

**Spasticity:** Increased tone of muscles. For example, with spasticity of the legs there is an increase in the leg muscles’ tone so they feel tight and rigid and the knee jerk reflex is exaggerated.

- None: Normal muscle tone and reflexes.
- Mild: Extremity muscles are tight and reflexes are abnormally increased, but only the patient and physician are aware of it, and the spasticity does not interfere with physical function.
- Moderate: Patient’s spasticity is evident to those who watch the patient walk, but it is relatively subtle.
- Severe: Patient’s spasticity is very obvious to those who watch the patient walk, but patient is able to ambulate fairly easily and carry out most physical functions.
- Extremely Severe: Patient’s spasticity is very obvious and interferes greatly with physical function.

**Gross Motor Impairment:** How much difficulty is the patient having with walking?

- None: Able to walk normally.
- Mild: Able to walk by self (without a walker or assistance from another person), but walks abnormally slowly and weakly.
- Moderate: Able to walk many steps, but only slowly and only with the help of a walker or an assisting person.
- Severe: Able to stand up (with or without assistance), but able to take only a few (2-4) steps and needs assistance from another person in order to take those few steps.
- Extremely Severe: Patient is bedridden. Unable to even stand-up by self, even with the support of an assisting person.

**Fine Motor Impairment:** How much difficulty does the patient have when using hands and fingers to do delicate tasks, like writing, using scissors, using eating utensils, or picking up tiny objects.

- None: No difficulty
- Mild: Mild difficulty.
- Moderate: Moderate difficulty.
- Severe: Has severe difficulty performing delicate tasks with hands/fingers.
- Extremely Severe: Unable to use hands/fingers for any delicate tasks.

**Hemiparesis:** Hemiparesis means weakness in an arm, or leg (or both), due to injury to the part of the brain that controls arm or leg motion and strength. To what extent does the patient have permanent weakness (residual weakness) in an arm or leg?

- None: No hemiparesis---i.e. no residual weakness
- Mild: Mild weakness. Not noticeable to others.
- Moderate: Moderate weakness, barely noticeable to others
- Severe: Severe weakness. Obvious to others, because walks with a limp or has obvious difficulty using arm.
- Extremely severe: Walks with an extremely severe limp, or unable to walk.

**Neurogenic Bladder**: Abnormal urinary bladder function caused by a problem with the nervous system. Spontaneous, uncontrolled nerve impulses to the bladder trigger spastic, unexpected bladder contractions, resulting in accidental voiding or excessively frequent need to urinate.

- None: Normal bladder function.
- Mild: Occasionally has abnormal bladder function, but just mild and intermittent.
- Moderate: Every day, the patient needs to urinate with abnormal frequency, but has no accidents.
- Severe: Patient constantly deals with urinary frequency and frequently has accidental voiding.
- Extremely Severe: Patient is unable to void on his/her own. Patient requires daily catheterization to evacuate the bladder.

**Slurred Speech:** To what extent has the patient been left with speech that is difficult for others to understand?

- None: Has normal speech.
- Mild: Speech is at least intermittently a little bit slurred.
- Moderate: Speech is constantly moderately slurred
- Severe: speech is constantly severely slurred
- Extremely Severe: speech is unintelligible.

**______________________________________________________________________________**

**Inner Ear Damage:**

**Hearing Loss:**

- None: Patient has not noted any hearing loss. Audiogram, if done, has been normal.
- Slight: Patient suspects slight hearing loss, slight muffling or distortion of sound; but an audiogram is normal.
- Mild: Mild hearing loss noted by patient and on audiogram.
- Moderate: Moderate hearing loss noted by patient and on audiogram. Hearing aids have been suggested, at least as an appropriate option.
- Severe: Severe hearing loss, using hearing aid. Cochlear implant has been recommended, or at least offered as an option.
- Extremely Severe: Patient has undergone Cochlear implantation.

**Tinnitus:** Tinnitus means “ringing” in the ears. To what extent does the patient have tinnitus?

- None: No tinnitus.
- Mild: Patient has tinnitus, but is largely oblivious to it. Usually doesn’t notice it
- Moderate: Fairly bothersome tinnitus, but quite tolerable.
- Severe: Loud tinnitus. Barely tolerable.
- Extremely Severe: Has constant very loud, “roaring tinnitus” that is intolerable and “driving patient crazy.”

**Dizziness/Vertigo:** To what extent does the patient have a sensation of spinning or whirling—a sensation of objects rotating about the patient, or of the patient rotating about objects?

- None: No vertigo.
- Mild: Occasional mild vertigo, but able to function almost normally.
- Moderate: Moderate vertigo when upright. Functional only while in supine position.
- Severe: No vertigo while supine; severe vertigo when sits up or stands. Largely incapacitated.
- Extremely Severe: The whirling sensation is so severe and constant that the patient is constantly nauseated, frequently vomiting, and unable to assume anything other than a motionless supine position. Totally incapacitated by the vertigo.

**Extent to which Hearing Loss is Adversely Affecting QOL:** On a scale of zero to 100, indicate the extent to which any hearing loss you have has diminished the quality of your life. **Zero** would mean that either you do not have any hearing loss, or any hearing loss you have has not, in your opinion, diminished the quality of your life at all. **100** would mean that your hearing loss has extremely severely diminished the quality of your life.

**_____________________________________________________________________________________**

**Eye Damage:**

**Permanent Blind Spot:** The purpose of this question is to document the size of any permanent (fixed) scotoma/scotomas (blind spots) that you are noticing---expressed as the percentage of your visual field that you are missing because of one or more blind spots (scotomas). If you have no blind spots, then you are missing 0% of your visual field. If you have two permanent blind spots in your right eye, one blocking 20% of your visual field in that eye and the other blocking 15% in another part of the visual field of that eye, then these two scotomas have caused you to lose 35% of your visual field in the right eye. So, you would enter 35.

**Constricted Peripheral Vision:** The purpose of this question is to document (on a scale of 0-100) the extent to which you have noticed loss of peripheral vision---none, mild, moderate, severe, or extremely severe loss of peripheral vision. If you have severe “tunnel vision” (like looking through a toilet paper tube), you would have extremely severe loss of peripheral vision (no peripheral vision).

**___________________________________________________________________________________**
